# Manipulating cavity photon dynamics by topologically curved space

**DOI:** 10.1038/s41377-022-01009-x

**Published:** 2022-10-25

**Authors:** Yongsheng Wang, Yuhao Ren, Xiaoxuan Luo, Bo Li, Zaoyu Chen, Zhenzhi Liu, Fu Liu, Yin Cai, Yanpeng Zhang, Jin Liu, Feng Li

**Affiliations:** 1 Key Laboratory for Physical Electronics and Devices of the Ministry of Education & Shaanxi Key Lab of Information Photonic Technique, School of Electronic Science and Engineering, Faculty of Electronic and Information Engineering, Xi’an Jiaotong University, Xi’an, 710049 China; 2 State Key Laboratory of Optoelectronic Materials and Technologies, School of Physics, Sun Yat-sen University, Guangzhou, China

**Keywords:** Microresonators, Photonic devices

## Abstract

Asymmetric microcavities supporting Whispering-gallery modes (WGMs) are of great significance for on-chip optical information processing. We establish asymmetric microcavities on topologically curved surfaces, where the geodesic light trajectories completely reconstruct the cavity mode features. The curvature-mediated photon-lifetime engineering enables the enhancement of the quality factors of periodic island modes by up to 200 times. Strong and weak coupling between modes of very different origins occurs when the space curvature brings them into resonance, leading to fine tailoring of the cavity photon energy and lifetime and the observation of non-Hermitian exceptional point (EP). At large space curvatures, the role of the WGMs is replaced by high-Q periodic modes protected by the high stability of island-like light trajectory. Our work demonstrates interesting physical mechanisms at the crosspoint of optical chaotic dynamics, non-Hermitian physics, and geodesic optical devices, and would initiate the novel area of geodesic microcavity photonics.

## Abstract (web summary)

We show that the photon dynamics and lifetimes in asymmetric optical microcavities are greatly modified if the cavity is constructed in a topologically curved space.

## Graphical abstract

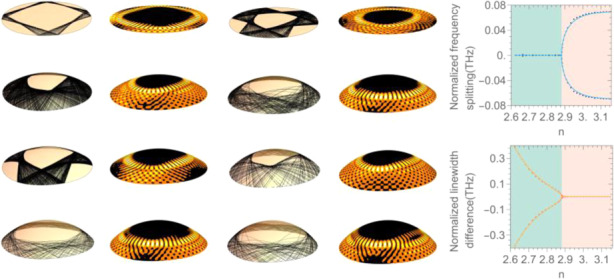

## Introduction

Optical microcavities supporting whispering-gallery-mode (WGM) are resonators confining light via successive total internal reflections (TIR) that are extensively investigated for applications in on-chip microlasers^1–3^, sensors^4–7^, filters^8^, isolators^9,10^, frequency combs^11,12^, and gain-loss featured optoelectronic devices^13–15^. The cavity periphery basically exhibits a circular or polygonal shape in the forms of microdisks, microspheres, microtoroids, microwires, and microtubes^16–22^. To achieve particular features such as unidirectional lasing, asymmetric cavities were designed and fabricated by either abruptly or smoothly deforming the cavity periphery^23–25^, which leads to stable and chaotic optical modes^26–28^. The chaotic motion of photons in the cavity is usually analyzed by ray dynamics which records the reflecting angle as a function of the reflecting position in a diagram called the Poincare surface of section (PSOS)^29–32^ and then compared with its counterpart of simulations using wave optics. It is shown that the chaotic modes in deformed cavities are particularly useful to achieve broadband resonance and nonlinear effects such as frequency combs^29,33,34^. Meanwhile, controlling the gain and loss of the microcavity modes leads to non-Hermitian physics, which provides a new mechanism for improving device performances^5,8,14,28,35,36^.

Recently, curved membranes are developed to form three-dimensional (3D) on-chip photonic structures, which would allow the much higher capacity of devices on integrated photonic circuits compared to the two-dimensional (2D) counterparts^37,38^. Interestingly, it has been experimentally demonstrated that a 2D microdisk cavity can be bent up to support WGMs exhibiting 3D trajectories in space, while keeping a considerably high-quality factor^39,40^. It is therefore interesting to consider if a WGM defined on a curved 2D surface can be efficiently analyzed with both ray dynamics and wave optics, and most significantly, if such structures bring fundamental changes that would potentially lead to a great improvement of device performance and on-chip integration.

In this article, we investigate the nature of photonic modes in asymmetric microcavities defined on a topologically curved surface, by modeling the light traces as geodesic lines. We find that the cavity photon energy and lifetime can be continuously tailored by varying the 2D space curvature, leading to a giant increase of quality factors (Q-factor) of the periodic island and chaotic modes up to ~200 times. The space curvature brings modes of very different origins into resonance, resulting in mode hybridization in the strong and weak coupling regimes. By tuning the overall loss, the non-Hermitian exceptional point (EP) is reached at which the bifurcations of both the photon energy and lifetime vanish. Particularly, at large space curvatures, the high-Q WGMs vanish while being replaced by the highly-hybrid periodic modes whose low-loss property is protected by the stability of the island-like trajectories. Our results reveal a series of novel physical effects created by the space curvature combining the advances of optical chaotic dynamics, geodesic photonics, and non-Hermitian physics, which would open the area of geodesic microcavity photonics for the improvement of device performance.

## Results

**Fig. 1:**
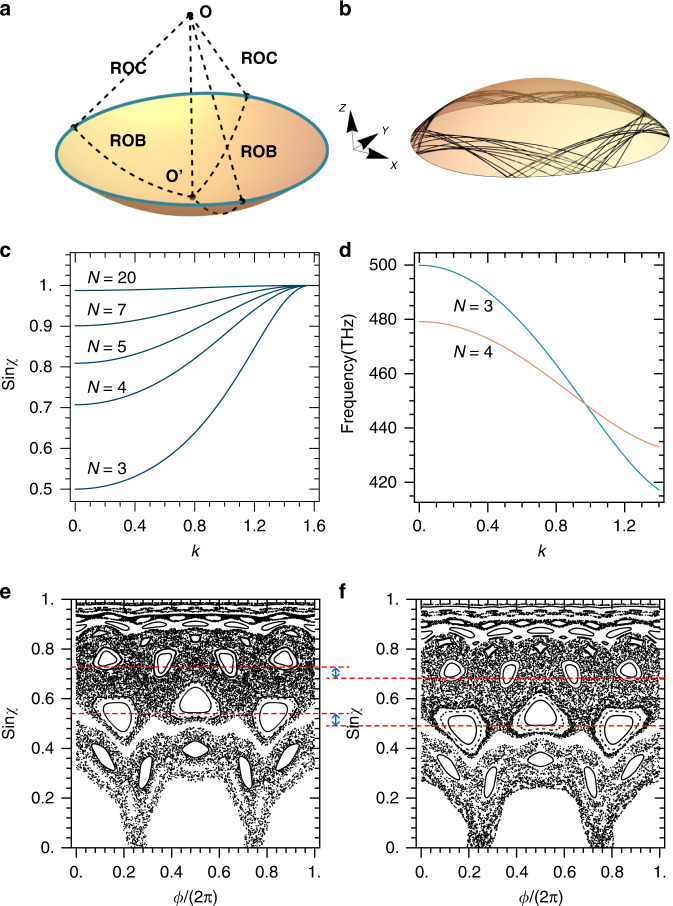
Ray dynamics of topologically curved microcavities. **a** Schematic picture of the microcavity. O and O’ are the center of the spherical surface and the geometric center of the cavity, respectively. ROC: radius of curvature; ROB: radius of the boundary. **b** Light trajectories of a symmetric circular cavity with ROB = 0.8 defined on a curved surface of ROC = 1. **c** sin*χ* (*χ* is the reflecting angle) of the N-period modes as a function of the effective curvature *k* = ROB/ROC in a symmetric circular microcavity. **d** Calculated resonant frequencies for the 3-period (red) and 4-period (blue) modes as a function of *k* in a symmetric circular microcavity. **e**, **f** The PSOS of a Face cavity defined on a curved surface with *k* = *π*/6 (**e**) and on a flat surface (**f**). The dashed horizontal lines label the bottoms of the higher islands in the 3-period and the 4-period groups, which correspond to identical structural positions in the PSOSs of **e**, **f**, showing a discrepancy in the height of sin*χ*

In a curved space, the traditional ray dynamics assuming straight light trajectories no longer applies, and the proper light trajectories are the geodesic lines determined by the space metric tensor, ensuring the optical length of the extremum it passes^41^. In comparison with flat 2D surfaces, curved 2D surfaces can be classified into two categories: the ones that can and cannot be unfolded to become flat surfaces. The former, such as the bent-up microdisks^39,40^, is not of our interest as its geodesic lines exactly correspond to the straight lines on the unfolded plane, making no difference in ray dynamics compared to a flat surface. Nevertheless, the latter is much more interesting, as it is formed based on a general definition of topological deformation from a flat surface by keeping the topological invariant, leading to the reconstruction of light trajectories. Herein the concept of “topologically curved” is purely geometrical, referring to shapes that cannot be unfolded along any direction, instead of claiming topological phases in physics. It is sufficient to demonstrate the dramatic effects of such topological deformations by investigating a WGM cavity defined on a spherical surface, whose geodesic line is well known to be the great circle. Figure 1a shows an illustrative picture of the cavity, in which the ROC presents the radius of curvature of the spherical surface, and the ROB represents the radius of the boundary of the cavity, defined as the distances between the cavity boundary and its geometric center along the geodesic lines. The PSOS is derived by tracing the light trajectory as geodesic lines reflecting at the cavity boundary. (see Materials and methods). Obviously, cavities with the same ratio *k* = ROB/ROC have exactly the same geometric shape, as the PSOS is irrelevant to the absolute size of the cavity. Therefore, we define *k* as the effective curvature, which is the most crucial parameter affecting the ray dynamics. An example of the calculated light trajectories in a symmetric circular cavity with *k* = 0.8 (ROC = 1, ROB = 0.8) is shown in Fig.1b.

The general influence of space curvature on ray dynamics can be simply evaluated in symmetric circular cavities, which support regular WGMs whose light trajectories form inscribed regular polygons consisting of geodesic lines. The sine of the reflecting angles (noted as sinχ) of the WGMs associated with *N*-sided polygons is plotted as a function of the effective curvature *k* in Fig. 1c. It is clearly seen that sinχ increases with *k*, originating simply from the nature of the spherical surface on which the inner angle of a regular polygon is larger than that of its flat counterpart. Particularly, at *k* = 0 (ROC is infinite) the cavity reduces to its flat counterparts, and at the extreme case of *k* = *π*/2, where the cavity becomes a surface of the hemisphere with its boundary itself being a great circle, the WGMs reach a special regime where the trajectories of *N* = 2 WGMs can exhibit arbitrary reflection angles and the trajectories of all the *N* > 2 WGMs must be the cavity boundary itself, indicating a definite condition of ${{{\mathrm{sin\chi }}}} = 1$. In this work, we constrain our studies in the regime of 0 < *k* < *π*/2 to ensure reflections at the cavity boundary always happen, in accordance with the definition of curved WGM cavities.

The increase of sinχ with *k* indicates that the loss of the optical modes, especially those associated with small *N*, could be greatly reduced in curved space. Meanwhile, the space curvature leads to a variety of optical lengths of the round trip and thereby the resonant frequency. Figure 1d shows the calculated resonant frequencies for the 3-period (meaning light reflecting three times within one round trip, i.e., *N* = 3, and thereafter) and 4-period modes as a function of *k*, derived by letting the optical length of the round trip equal to an integer time of the optical wavelength, namely,

1 $$\nu = m \times \frac{c}{{n_{{\rm{eff}}}l}}$$

Where *ν* is the mode frequency, *n*_eff_ is the effective refractive index, *l* is the line length of the round trip given by the ray dynamics, *c* is the vacuum light velocity and *m* (integer) is the mode azimuthal order. The frequencies of the 3-period and 4-period modes exhibit different slopes of variation and become degenerate at *k* = 0.9722. This kind of behavior can lead to the coherent coupling between modes of different physical origins, allowing the modification of the real and imaginary parts of the eigenvalues of the optical resonances.

To test the idea in detail, an asymmetric cavity needs to be designed to allow stable *N*-period island modes with *χ* near or even below the TIR angle, the Q-factor of which would be very low if the cavity is flat. We choose the well-known “Face cavity”^23^ as an example, whose boundary is expressed by

2 $$R{{{\mathrm{ = }}}}\left\{ {\begin{array}{*{20}{c}} {R_0 \cdot (1 + a_2 \cdot \cos ^2\phi + a_3 \cdot \cos ^3\phi )} & {\phi \in \left( {\left. {\frac{{ - \pi }}{2},\frac{\pi }{2}} \right)} \right.} \\ {R_0 \cdot (1 + b_2 \cdot \cos ^2\phi + b_3 \cdot \cos ^3\phi )} & {\phi \in \left( {\left. {\frac{\pi }{2},\frac{{3\pi }}{2}} \right)} \right.} \end{array}} \right.$$

in which *ϕ* is the azimuthal angle with $\phi = 0$ pointing along the positive direction of the x-axis illustrated in Fig. 1a. $R_0,a_2,a_3,b_2$ and $b_3$ are the shape parameters. The only difference from the flat cavity is that herein *R* is the ROB measured along the geodesic line of the spherical surface. In our calculations we apply the shape parameters $R_0 = 1,a_2 = - 0.1329,a_3 = 0.0948,b_2 = - 0.0642,b_3 = - 0.0224$. The PSOS of the “Face” cavity defined on a spherical surface of *k* = *π*/6 and its flat counterpart is shown in Fig. 1e, f, respectively. Whilst the details of some islands (e.g., the 5-period ones) and the distribution of chaotic modes become different, the most obvious feature is that all the island and WGM modes are moved upwards by the space curvature to larger ${{{\mathrm{sin\chi }}}}$, compared to the flat cavity.

**Fig. 2:**
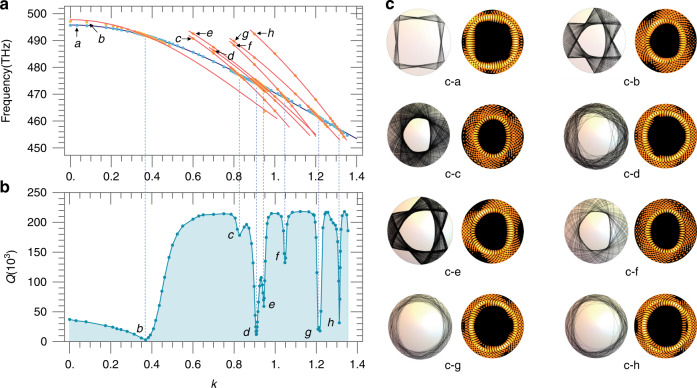
Features of various photonic modes in topologically curved microcavities. **a**–**h labels modes of different origins. a** The frequency of modes a-h as a function of *k*. **b** The Q-factor of mode a (which is a 4-period island mode) as a function of *k*, showing a series of dips corresponding to the resonant point with other modes (labeled with the corresponding letters). **c** Light trajectories [left panels of (**c**–**a**) to (**c**–**h**)] and electric field spatial profiles [right panels of (**c**–**a**) to (**c**–**h**)] of modes **a**–**h** (top view). Each graph in **c** corresponds to the point in **a** pointed by the arrow with the associated letter

We perform mode analysis with wave optics using COMSOL Multiphysics (Version 5.6). The cavity parameters are set to be refractive index ${\it{n}} = \sqrt {10} \approx 3.1622$ and the cavity layer thickness *d* =50 nm. In the simulations, we keep the value $R_C = ROC \ast {{{\mathrm{sin}}}}(k)$ a constant of $2.1 \,\upmu {\rm{m}}$ (which keeps the cavity circumference $2\pi R_C$ a constant for good comparability), while varying the value of *k* continuously from 0 to 1.4. We analyzed the 4-period island modes, the 3-period island modes, and their nearby chaotic modes in the PSOS, and plot their frequencies as a function of *k* as colored dots in Fig. 2a, in which the colored solid lines represent a coarse fitting of modes using the resonant conditions of Eq. (1). The modes are labeled by the letters a–h, with the spatial profiles of light intensity and light trajectories of ray dynamics shown in Fig. 2c. The graphs are all presented in a top-down view, whilst the 3D view is available in the Supplementary Information. It should be noted that even without the fitting lines, we can still easily identify and trace each mode of a-h thanks to the continuous variation of the mode frequencies and spatial features with *k*. By comparing the mode spatial profile, the light trajectories, and the distribution on the PSOS (see Supplementary Information), we can clearly identify that mode a is a 4-period island mode, modes b and e are 3-period island modes, and modes d, g and h (resp. c and f) are chaotic modes appearing around (resp. below) the 3-period island modes in the PSOS.

**Fig. 3:**
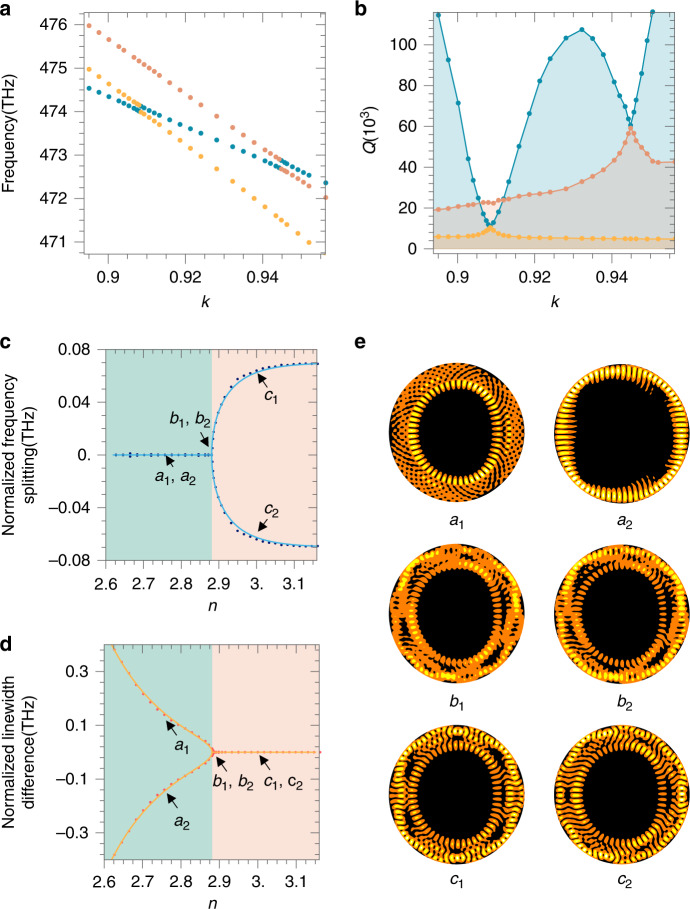
Geodesic mode coupling and non-Hermitian EP. **a** Mode frequencies as a function of *k*, showing anti-crossing features at each resonant point. **b** The Q-factor of mode a as a function of *k*. **c**, **d** The splittings of the real (**c**) and imaginary (**d**) parts of the eigenvalues of the hybrid modes formed by the coupling between modes **a** and **d**, as a function of the material refractive index *n*. **e** The electric field distributions (top view) of the corresponding points a_1_, a_2_, b_1_, b_2_ and c_1_, c_2_ in **c** and **d**. The degenerate points b_1_ and b_2_ correspond to the EP. Note here the letters a_1_ to c_2_ are used to label the points in this figure and are not associated with the labeling of different modes

With increasing *k*, the modes of different origins show different slopes of frequency variation, and cross each other at certain points where they coherently interact. Indeed, after plotting the Q-factor of the 4-period island mode a as a function of *k* in Fig. 2b, we observe sharp dips at the crossing points with the 3-period island and chaotic modes which are more lossy, indicating coherent coupling between modes. We perform further simulations with smaller footsteps at the crossing point between modes a, d and e, as shown in Fig. 3a, b. Instead of simply crossing each other, the modes show anticrossing feature with a splitting of frequency at the resonant points, i.e., *k* = 0.9081 for a–d and *k* = 0.9444 for a–e, where the Q-factors of the coupled two modes become identical. Frequency splitting and loss modification as a result of mode coupling has been investigated in WGM microcavities^5,27,42^ and nanostructures^43^, being a typical feature of the non-Hermitian system which can be described by the Hamiltonian in matrix form:

3 $$\left( {\begin{array}{*{20}{c}} {\omega _1{{{\mathrm{ - i}}}}\gamma _1} & \mu \\ \mu & {\omega _2{{{\mathrm{ - i}}}}\gamma _2} \end{array}} \right)$$

where *ω*_1,2_ and *γ*_1,2_ are the mode frequency (the real part of the eigenvalue) and linewidth (the imaginary part of the eigenvalue) of the uncoupled bare modes and *μ* is the coupling strength. At the resonance condition of $\omega _1 = \omega _2 = \omega$, the eigenvalues of the coupled system are

4 $$\sigma _ \pm = \omega - {{{\mathrm{i}}}}\gamma _{{{{\mathrm{ave}}}}} \pm \sqrt {\mu ^2{{{\mathrm{ - }}}}\gamma _{{{{\mathrm{diff}}}}}^2}$$

where ${{{\mathrm{\gamma }}}}_{{{{\mathrm{ave}}}}} = \frac{{\gamma _1 + \gamma _2}}{2}$ and ${{{\mathrm{\gamma }}}}_{{{{\mathrm{diff}}}}} = \frac{{\gamma _1 - \gamma _2}}{2}$. When $\mu ^2 - \gamma _{{{{\mathrm{diff}}}}}^2 \,> \, 0$ (resp. $\mu ^2 - \gamma _{{{{\mathrm{diff}}}}}^2 \, < \, 0$) the system is in the strong (resp. weak) coupling regimes featured by a bifurcation of the real (resp. imaginary) part of the eigenvalue, and the EP is the transition point where both the real and imaginary parts of the eigenvalue coalesce^14,44,45^. As the frequency and the Q-factor are associated with the real and imaginary parts of the eigenvalue respectively, the a-d and a-e mode couplings featured in Fig. 3a, b are both in the strong coupling regime. They can possibly turn into a weak coupling regime by modifying either the coupling strength $\mu$ and the difference in loss $\gamma _{{{{\mathrm{diff}}}}}$. Indeed, changing the refractive index *n* is an effective way to tune the difference in loss, as it has more influence on the lossy mode than the conservative mode. We perform mode analysis using COMSOL on a cavity of *k* = 0.9081 where the bare modes a and d are in resonance, and vary *n* with a small step size of 0.017. The results of the normalized frequency splitting (real part of $\frac{{{{{\mathrm{\sigma }}}}_ + - \sigma _ - }}{2}$) and the normalized linewidth difference (imaginary part of $\frac{{{{{\mathrm{\sigma }}}}_ + - \sigma _ - }}{2}$), shown as colored dots in Fig. 3c, d, exhibit bifurcation of the frequency (resp. the linewidth) when above (resp. blow) *n* = 2.88, indicating the strong (resp. the weak) coupling regimes. At *n* = 2.88, the non-Hermitian EP connecting the strong and weak coupling regimes is reached. These results can be well fitted by Eq. (4) with the parameter $\mu = 0.07$ THz, shown as the solid lines. Points (a_1_, a_2_) (b_1_, b_2_) and (c_1_, c_2_), which are in the weak coupling regime, at the EP and in the strong coupling regime are chosen to compare the spatial field distribution in Fig. 3e. Whilst exhibiting differences in both the weak and strong coupling regimes, the mode profiles are completely identical at the EP, indicating the achievement of a single eigenstate.

It is interesting to look back at the Q-factor of the 4-period island mode in Fig. 2b, in which the dips at the intersections of a–c and a–f are shallower than the others. This is because mode a is actually in weak coupling with the chaotic modes c and f, which exhibit the highest loss even at a high refractive index of $n = \sqrt {10}$, and the Q-factors of the coupled modes do not coalesce, resulting in smaller photon-lifetime modification. Nevertheless, the a-e dip, though also seems shallow, reflects the strong coupling owing to the higher Q associated with mode e which is a 3-period regular mode.

**Fig. 4:**
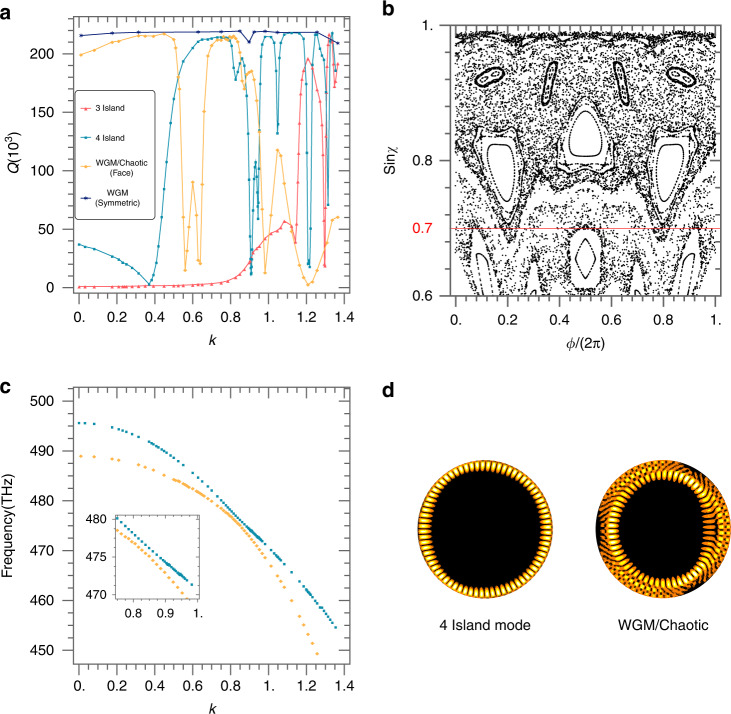
Curvature-mediated photon-lifetime engineering. **a** Q-factor vs. *k* for the 4-period (green), the 3-period (red), the WGM (yellow, which becomes a hybrid 4-period chaotic mode at *k* > 0.81) of the Face cavity and the WGM of a symmetric cavity (blue). **b** PSOS of the Face cavity with *k* = 1.16. **c** Mode frequency of the 4-period island and the WGM showing strong coupling to form hybrid modes. The inset is an enlarged view around the anticrossing point. **d** Spatial field distribution of the hybrid 4-period island (left) and WGM-chaotic (right) modes at *k* = 1.16

Then it comes to the essential question of how the photon lifetimes of different types of modes are engineered by the space curvature. The Q-factors of the 3 and 4-period island modes (modes a and b in Fig. 2) and the WGM of the Face cavity are plotted as a function of *k* in Fig. 4a, together with a WGM of a symmetric (perfect circular) cavity for comparison. If we ignore the dips caused by mode coupling, the Q-factor of the 4-period island mode generally increases with *k* up to a value of ~220,000, which is 4–5 times larger than the same mode of a flat Face cavity (~50,000), whilst the increase becomes ~200 times for the 3-period island mode, from *Q* =~ 1000 of the flat cavity to also ~220,000 at large *k*, demonstrating the unique merit of the space curvature. Nevertheless, there is almost no increase in Q for the WGMs. The reason for the different quantities comes from the wave-like nature of light. Even if the reflection angle ${{{\mathrm{\chi }}}}$ is larger than the TIR angle (${{{\mathrm{sin\chi }}}} = \sim 0.316$ for our cavity) when analyzed using ray dynamics, the actual light wave, presenting non-zero size and divergence angle, still contains a portion of which the sinχ does not satisfy TIR and causes loss. Therefore, the Q-factor of a mode increases rapidly with its ${{{\mathrm{sin\chi }}}}$ (and thereby with *k*) until reaching a quasi-saturating point far above the TIR line at which the wave optics loss reduces to a certain limit and high Q-factor is obtained. In our specific Face cavity, this point is demonstrated to be at ${{{\mathrm{sin\chi }}}} = \sim 0.7$, corresponding to *k* = ~0.6 for the 4-period and *k* = ~1.2 for the 3-period island modes, at which the increases of Q nearly saturate (associated PSOS graphs in Fig. S2 of the Supplementary Information). Contrarily, the sin*χ* of the WGMs is already much larger than 0.7 at *k* = 0, leading to a slow post-saturation increase of Q. The common maximum value of Q, which is ~220,000 in the studied cavity, corresponds to the intrinsic Q-factor of the high-Q WGMs in flat cavities, excluding the effect of mode coupling.

Interestingly, the high-Q WGMs cease to exist when the periodic islands are lifted to a position sufficiently high in the PSOS. As shown in Fig. 4b, at *k* = 1.16, the line of WGM “breaks” to become completely chaotic, while the 4-period islands remain existing but with their size and shape changed. Strong hybridization between the two modes occurs, with a coupling constant (*μ* = 0.5615 THz) almost one order of magnitude higher than in Fig. 3, as shown in Fig. 4c. The resulting new eigenmodes include a 4-period island and a 4-period chaotic mode, shown in Fig. 4d, displaying high and low Q-factors (Fig. 4a at the range of *k* > 0.8**)**, respectively, whilst their spatial profile evolution with *k* are plotted in Fig. S3 of the Supplementary Information. Although the ray dynamics displays an equivalent range of ${{{\mathrm{sin\chi }}}}$ for the two modes, a large discrepancy in Q is present. The high Q of the island mode results from its high stability, which protects it from strong tunneling through chaotic channels to leaky regions, as already proved experimentally in ref. ^46^, while the low Q of the chaotic mode is due to its low stability against strong dynamic tunneling. The results reveal the important physical mechanism that the high-Q modes in highly-curved asymmetric cavities are stability-protected island modes instead of the WGMs.

**Fig. 5:**
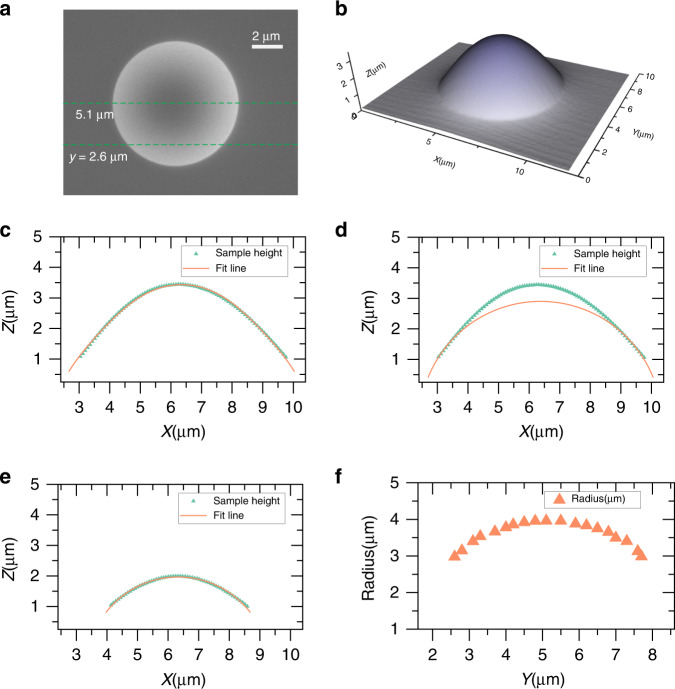
Structural property of the fabricated curved surface on GaAs substrate and measurement. **a** top view of the SEM image, in which the green dashed lines label the position of the sections in **c**–**e**, in which *y* = 5.1 μm corresponds to the diameter of the surface. **b** The AFM image shown in 3D form. **c** Fit of the surface of the section at *y* = 5.1 μm from the AFM image in **b**, with a circle graduate changing its radius with the height axis Z. The fit line follows the equation $\left( {x - x_c} \right)^2 + \left( {z - z_c} \right)^2 = R^2\left( {1 + a \cdot \sin \theta + {{{\mathrm{b}}}} \cdot \sin ^2\theta } \right)^2,\tan \theta = (z - z_c)/(x - x_c)$, where ${{{\mathrm{x}}}}_{{{\mathrm{c}}}} = 6.35\,{{{\mathrm{\mu m}}}},z_c = 0.56507\,{{{\mathrm{\mu m}}}},a = - 0.62026,b = 0.39503,R = 3.70538\,{{{\mathrm{\mu m}}}}$. **d** Circle fit of the same data in **c**, but with the equation $\left( {x - x_c} \right)^2\, +\, \left( {z - z_c} \right)^2 = R^2$, where $x_c = 6.37678\,{{{\mathrm{\mu m}}}},z_c = - 1.06306\,{{{\mathrm{\mu m}}}},R = 3.96216\,{{{\mathrm{\mu m}}}}$. The fitting works well for the r**e**gion of *z* = 1~2 μm, in which the WGM and periodic modes actually forms. **e** Circle fit of the section at *y* = 2.6 μm of the AFM image in **b**. The fit line follow the equation $\left( {x - x_c} \right)^2 \,+\, \left( {z - z_c} \right)^2 = R^2$, where $x_c = 6.32893\,{{{\mathrm{\mu m}}}},z_c = - 1.01123\,{{{\mathrm{\mu m}}}},R = 2.97804\,{{{\mathrm{\mu m}}}}$; **f**. The radii of the fit circles, obtained similarly as in **d** and **e**, as a function of the cross-section point on the *Y* axis

The experimental realization of the curved microcavities requires the fabrication of the curved spherical surface with sharp boundaries on semiconductor materials. We have developed a fabrication method enlightened by the typical process flow of semiconductor microlenses^47^. The spherical surface is first defined by a reflowed photoresist initially shaped with e-beam lithography (EBL) on a GaAs substrate, and then transferred to the GaAs substrate by inductively coupled plasma (ICP) etching. Details of the fabrication process is described in the Supplementary Information, and the morphology of the sample surface is shown in Fig. 5, including the scanning electron microscope (SEM) and atomic force microscopy (AFM) images in Fig. 5a, b, respectively. The geometry of the surface can be well fitted with a spherical one gradually changing its radius with the height coordinate, as shown in Fig. 5c, which should exhibit qualitatively the same features of photon dynamics as the single spherical surface. Meanwhile, the surface shape can be fitted by a circle of fixed radius at the height range of 1~2 μm, as demonstrated by Fig. 5d–f, where the WGM and periodic modes are actually located on the curved microcavity that is to be further fabricated. In addition, the surface exhibits sharp circular boundary displayed by the AFM and SEM (see Supplementary Information for side-view SEM images), which is a necessity to define the ROB for the investigated microcavitiy structure, contrary to the standard microlens which displays a boundary-less Gaussian shape^47^. The photoresist could be designed to have asymmetric shapes during the lithography to allow asymmetric surface boundaries. Meanwhile, the size of the structure, up to a dimeter of >8 μm, is 6–8 times larger than the standard microlens, making it suitable for WGM microcavities at optical wavlengths. There are several other reported methods for fabricating 3D curved structures, such as direct laser writing for Mobius trip microlasers^41^, 3D printing for geodesic lenses^48,49^, and focused ion beam etching for microscale concave mirrors^50^, which are also promising techniques for fabricating the curved cavity. In comparison, the EBL-reflow-ICP method adopts standard nanofabrication technologies on inorganic semiconductor substrates without deteriorating the optical properties of the material, which is essential for achieving semiconductor microlasers compatible with other on-chip photonic devices. Several further crucial steps are still to be carried out for the experimental demonstration of the physical effects in the curved cavities. The height range of 0~1 μm is to be replaced by an epitaxial sacrificial layer which is to be removed selectively for achieving a free-standing curved surface, and suitable optical characterization setups for detecting the specific optical modes are to be established. These complicated works are planned for the future and are out of the scope of this paper.

## Discussion

In this article, we investigated the dynamics of optical modes in topologically curved asymmetric microcavities, and reveals a series of novel physical effects including curvature-mediated lifetime control, EPs associated with geodesic coupling, and stability-protected high-Q islands. We notice that the work is only based on the simplest shape of curvature, implying that much richer microcavity physics with space curvature is to be explored in the future. The curvature-mediated approach provides an extra degree of freedom to tailor the resonant conditions, photon paths^51^, loss channels, and 3D emissions, which would be useful for constructing the next generation of optoelectronic devices such as microdisk lasers of controlled 3D directional emission, microcavity sensors of higher-order EPs, and 3D on-chip optical filters and couplers. The curved space-time in a confined system could also be potentially interesting for optical simulators of astrophysics and cosmology^52–54^. The research area of geodesic microcavity photonics, initiated by this study, would significantly contribute to new physics and optoelctronic applications.

## Materials and methods

### The algorithm of ray dynamics on a curved surface

We regard the curved 2D surfaces as very thin waveguides in which light travels with a uniform effective refractive index *n*_eff_. Therefore, like traveling in straight lines in flat 2D cavities, light ray travels in geodesic lines in curved space, which ensures the optical length of extremum it passes. The light trajectory of geodesic line intersects with the cavity boundary, and reflects at the intersecting point back into the cavity following the law of reflection, i.e., equal incident and reflecting angle with the tangent of the boundary. The algorithm thus contains 6 steps:

1. Choose an arbitrary starting point *P*_*0*_ at the cavity boundary (which is generally a spatial curve C) and an arbitrary initial traveling direction ***l***_0_ (a tangent vector of the curved cavity surface Σ at *P*_0_);
2. Derive the equation of the geodesic line *S* with *P*_0_ and ***l***_0_. *S* is then the trajectory of the incident beam.
3. Derive the intersecting point *P* between the incident beam *S* and the cavity boundary *C*, by simultaneously solving the equations of both. *P* is then the reflecting point.
4. Derive the tangent vector of *S* at *P*, noted as vector ***l***.
5. Derive the tangent vector of *C* at *P*. noted as vector ***t***. The angle formed by ***l*** and ***t*** is therefore the complementary angle of the incident angle at *P*.
6. Derive the tangent vector of the reflected beam at *P*, noted as ***l***^***’***^, following the law of reflection that the incident and reflecting angles are equivalent (so are their complementary angles), i.e., ${{{\boldsymbol{l}}}}^\prime \cdot {{{\boldsymbol{t}}}} = {{{\boldsymbol{l}}}} \cdot {{{\boldsymbol{t}}}}$. Meanwhile, the reflected light has to be inside the cavity surface Σ, i.e., ***l***^***’***^ has to be tangent to Σ, which requires ${{{\boldsymbol{l}}}}^{\prime} \cdot {{{\boldsymbol{n}}}} = 0$, where ***n*** is the normal vector of Σ at *P*. For spherical surfaces, ***n*** is the unit vector along the radial directions of the sphere.
7. Using *P* and ***l’*** as the new starting point and initial direction vector, run steps (2) –(6) repeatedly. After enough times of iterations, choose again a new set of *P*_0_ and ***l***_**0**_ for a new round of calculation.

In the algorithm, all tangent and normal vectors can be calculated using standard mathematical tools of vector analysis. Although the derivation of geodesic line equations, in general, requires tensor analysis (thereby differentially geometry), for the specific situation of regularly shaped surfaces like the spherical ones, existing solutions of mathematical expressions are available, i.e., the great circle. In each iteration of the algorithm, the position *P* and direction vector ***l*** are recorded. The collection of ***l*** vs. *P* constitutes the PSOS which is the standard analyzing method also for flat 2D cavities.

### The COMSOL simulation

The simulation is performed in a 3D cylindrical space with a radius of 3.6 μm, a height of 5.1 μm, a background refractive index of 1 (air), and absorbing boundaries. The simulation mesh is generated by the software, showing ~300 grids around the cavity periphery. In all simulations, the complex refractive index of the cavity material has a small imaginary part of $- 7.9056 \times 10^{ - 6}$. Each mode is double degenerate due to the clockwise and anti-clockwise propagation, and we choose one of them for analysis.

## Supplementary information

Supplementary

## Data Availability

The datasets generated and analyzed during the current study are available from the corresponding author upon reasonable request.
