# Supplementary material for: Manipulating cavity photon dynamics by topologically curved space
Source: Light Sci Appl. 2022 Oct 25;11:308. doi: 10.1038/s41377-022-01009-x (PMC9592597; doi:10.1038/s41377-022-01009-x)
Supplement: Supplementary file 1 — Supplementary [file 41377_2022_1009_MOESM1_ESM.pdf]

Supplementary information for “Manipulating cavity photon dynamics by topologically curved space”

Yongsheng Wang[1], Yuhao Ren[2], Xiaoxuan Luo[1], Bo Li[1], Zaoyu Chen[1], Zhenzhi Liu[1], Fu Liu[1], Yin Cai[1], Yanpeng Zhang[1], Jin Liu[2] and Feng Li[1]

[1]Key Laboratory for Physical Electronics and Devices of the Ministry of Education & Shaanxi Key Lab of Information Photonic Technique, School of Electronic Science and Engineering, Faculty of Electronic and Information Engineering, Xi'an Jiaotong University, Xi'an 710049, China

[2]State Key Laboratory of Optoelectronic Materials and Technologies, School of Physics, Sun Yat-sen University, Guangzhou, China

1. PSOS graphs and side-viewed ray trajectories and wave simulations of modes.

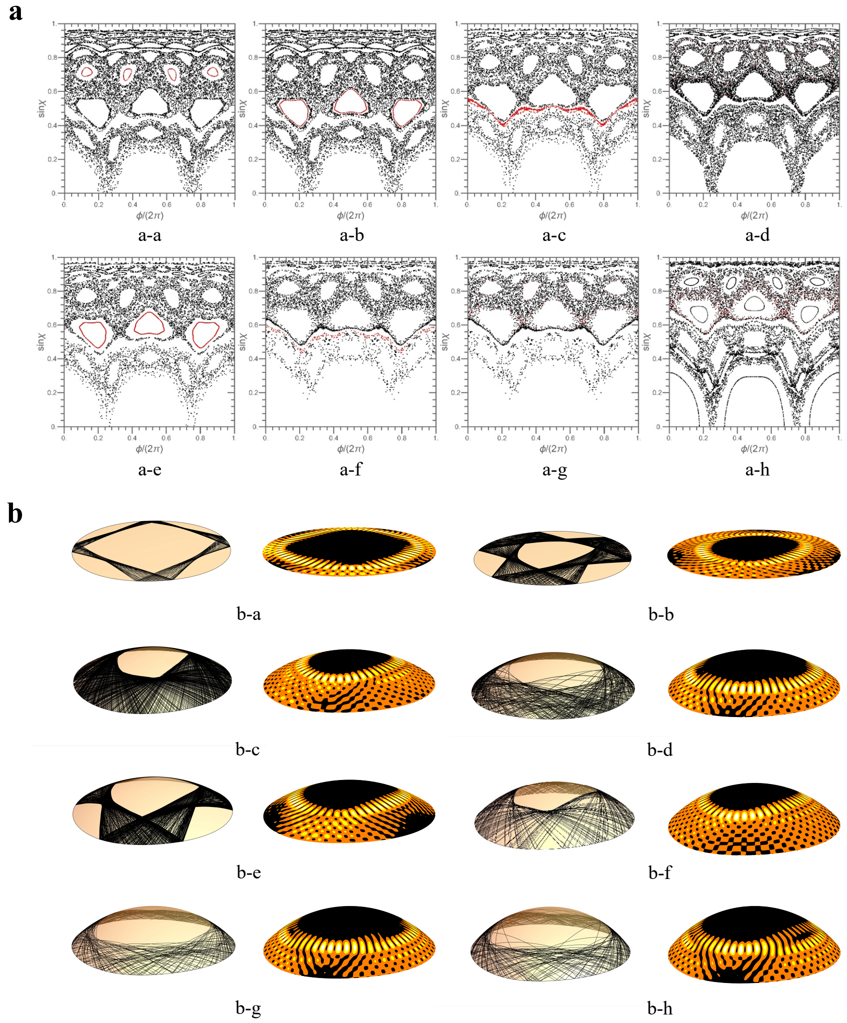

Fig. S1： Ray Dynamics and wave optics simulations of modes a-h in Fig. 3 of the main text. **a** The PSOSs of the curved microcavities supporting modes a-h [graphs from a-a to a-h], while the points corresponding to the trajectories in Fig. 3**c** of the main text are colored red, showing clearly the origin of each mode. **b** The side views of the light trajectories [left panels of b-a to b-h] and electric field

distributions [right panels of b-a to b-h] of modes a-h.

2. Curvature-mediated photon lifetime engineering.

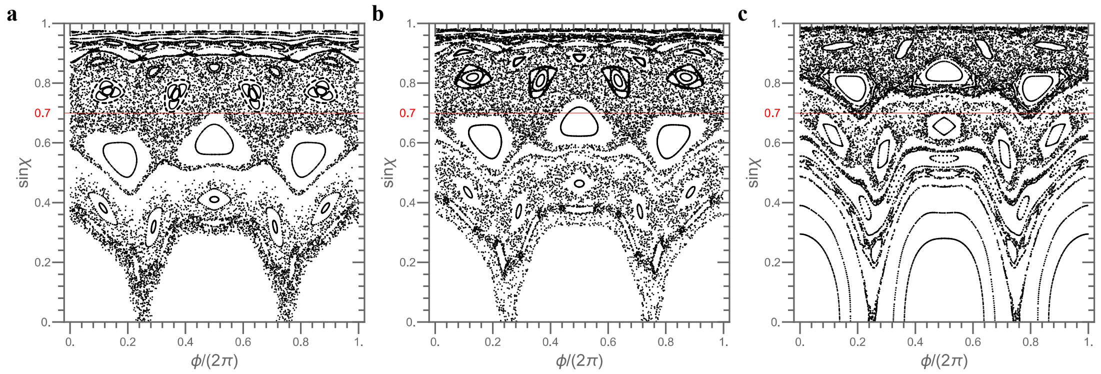

Fig. S2： PSOSs of the Face cavity for k=0.6 (**a**), 0.8(**b**) and 1.1636 (**c**). The red horizontal line represents sinχ=~0.7. It can be seen the shape of the islands (e.g. the 4-period) can change when they are pushed upwards to higher sinχ by the space curvature.

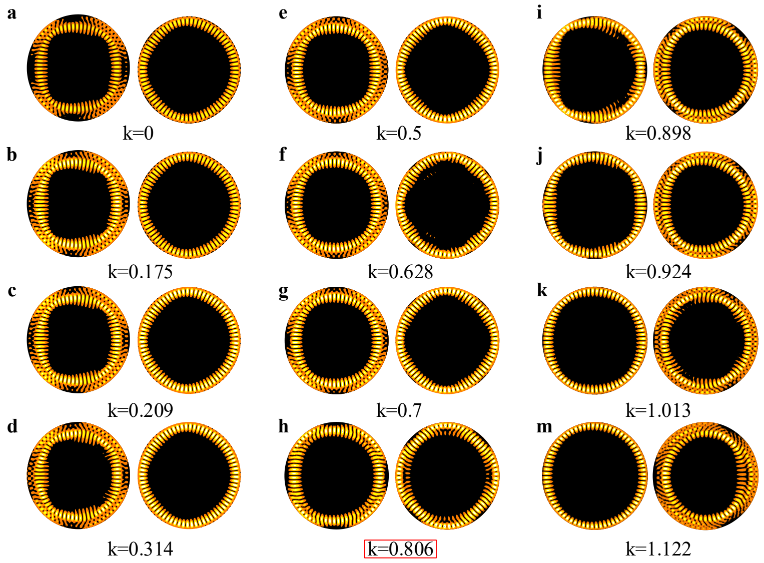

Fig. S3： Mode spatial profiles of the 4-peirod island (left) and the WGM-chaotic (right) modes at different values of *k*, while k=0.806 corresponds to the situation where the two bare modes are in resonance.

3. The fabrication of curved surface.

The sample of the curved surface is fabricated in 5 steps, which is illustrated in Fig. S4.

(1) The GaAs substrate is cleaned with acetone, isopropanol and deionized water for 10min, and bake at 150 °C for 10min after the cleaning.

(2) Spin coat the substrate with MaN-2410 photoresist at a speed of 3000 rpm, and bake at 90°C for 2min, reaching a thickness of about 1 micron.

(3) Define the circular-shaped pattern using electron beam lithography (EBL), and then develop with MaD-525 to transfer the pattern to the photoresist.

(4) Perform the reflow process: bake the sample at 160 ℃ for 5min after the development, and the photoresist melts into a hemispherical shape on the substrate surface.

(5) Etch the sample using ICP until the photoresist is just fully removed. The shape and morphology of the photoresist is then transfer onto the GaAs substrate. The resulting substrate displays a morphology of spherical surface, with a sharp circular boundary.

The SEM images of the sample is shown in Fig. S5 and Fig. S6.

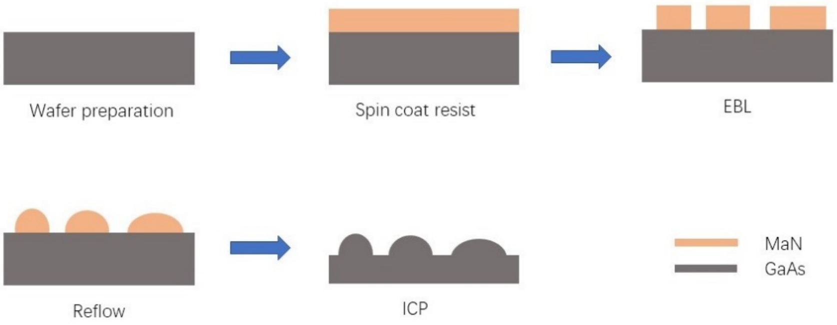

Fig. S4： Fabrication process of the curved surface on GaAs substrate.

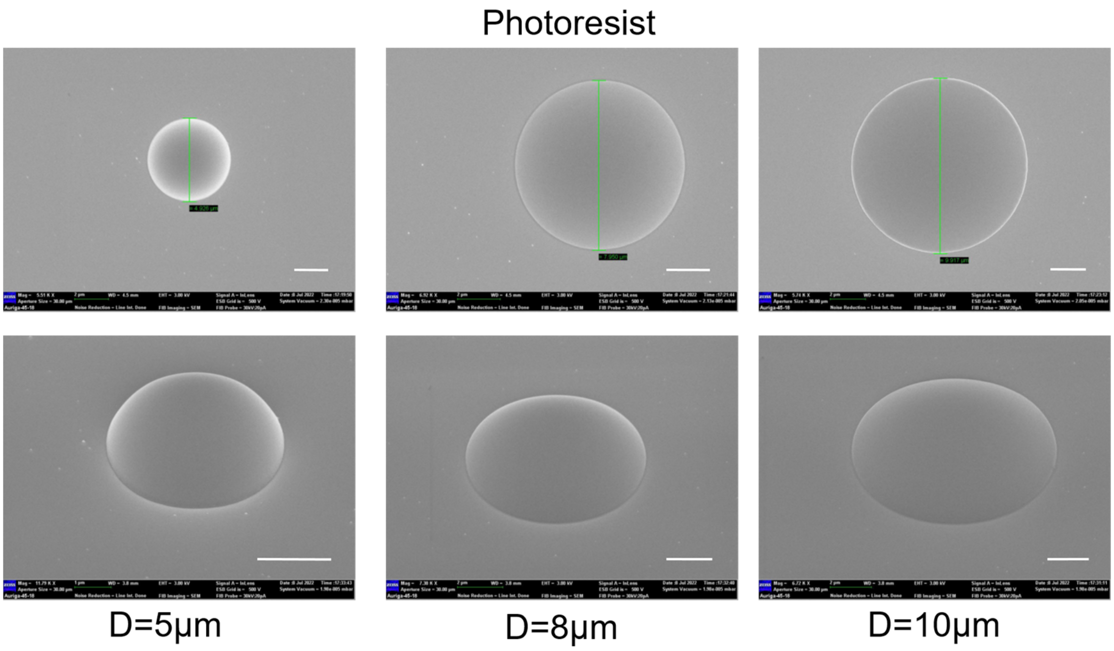

Fig.S5: The photoresist morphology after reflow (left to right: photoresist diameter of 5μm, 8μm, and 10μm), top view (upper panels) and the 45° side view (lower panels). The white horizontal bars in all figures represent 2μm.

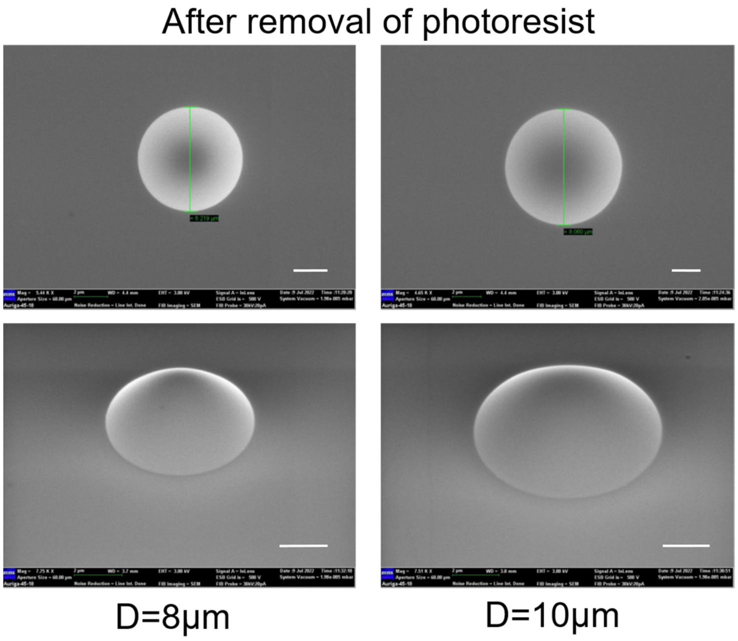

Fig.S6: The structure morphology after removing the photoresist with ICP etching (left to right: photoresist diameter of 8μm and 10μm, which yields 6.2μm and 8μm in diameter after the ICP etching), the top view (upper panels), and the 45° side view (lower panels). The white horizontal bars in all figures represent 2μm.
